# Editorial: Wishful thinking

**DOI:** 10.1186/1742-5573-1-2

**Published:** 2004-09-06

**Authors:** George Maldonado, Carl V Phillips, on behalf of the editors of Epidemiologic Perspectives & Innovations

**Affiliations:** 1 University of Minnesota School of Public Health, 420 Delaware St. SE, Minneapolis, Minnesota 55455 USA; 2 University of Texas Health Science Center, Houston, USA; 3 Center for Philosophy, Health, and Policy Sciences, Inc., Houston, USA

## Abstract

As a supplement to our lead editorial, the editors of the new journal, *Epidemiologic Perspectives & Innovations*, provide a partial list of specific analyses and topic areas they would like to see submitted to the journal.

## 

In our lead editorial [1], the editors of *Epidemiologic Perspectives & Innovations *(*EP&I*) present the underlying goals and philosophy of the journal, along with the types of articles we hope to publish. Here we clarify our goals with some specific ideas about articles we would like to see submitted to *EP&I *and with a "wish list" of methodological research and selected other innovations and perspectives we believe the field needs.

This list is far from complete, and we want to emphasize that our intention is not to discourage submissions on topics not specifically mentioned here. *EP&I *welcomes submissions of any papers in and of epidemiology, with the exception of those that solely report research results. "In and of" epidemiology includes papers about epidemiology and those grounded in other disciplines that might be useful for epidemiology. Many of our articles will be highly quantitative, but non-quantitative analyses are also welcome. We welcome submissions whose premises about how the field should be advancing differ from those of this editorial.

## Bridging the gap

*EP&I *hopes to help bridge the gap from best-practice methods to epidemiologic practice and teaching, and from there to epidemiology-based decision making.

There is a gap between what methodologists know about doing epidemiology and standard epidemiologic practice. We believe that narrowing this gap is as important as the development of new methodological ideas. Papers that would narrow this gap are encouraged. These could take different forms, including: clear presentations of advanced methods that are underutilized (perhaps written as tutorials or applications), surveys of epidemiologic practice to assess where the gaps might be, papers that function as advanced textbook chapters, and teaching articles. We will give the highest priority to papers that achieve excellence in explanation.

On a similar note, we would like to publish methods articles that fall into the category we call, "Don't Reinvent the Wheel." Other fields have different sets of methods that could be useful to epidemiologists, including observational methods from econometrics and related social sciences, uncertainty analysis methods from engineering and business, and historical perspectives from sciences with a longer history.

Also on this theme, we plan to commission updates to classic methods papers from the original authors. Methodological knowledge is ever advancing, and it would be interesting and useful to see what changes an author would make to a must-read paper if that paper were written today. We welcome suggestions of candidates for this series.

At the other end of the bridge, we invite methods and teaching papers that show how quantitative health researchers can present results that are more decision-relevant. We are especially interested in methods that go beyond statistical measures that emphasize merely whether an elevated risk has been found to ones that emphasize how much we ought to care about that elevated risk.

## New methodology

The editors of *EP&I *have identified several specific methodological areas that we believe need attention. Without intending to restrict methodology submissions to these topics, we are particularly interested in submissions that deal with: (1) full and proper disclosure of uncertainty in study results, (2) decision-making in the face of this full disclosure, (3) identification and control of confounding, (4) quantifying the effect of random error on study results, and (5) specification error (i.e., artifacts of statistical methods).

Papers that fall into category (1) have begun to appear in the epidemiologic literature [2-11] but much work remains to be done. For example, we especially encourage papers that deal with the specification of probability distributions for uncertainty-model parameters ("priors"), dependencies between priors, and uncertainty-analysis models for complicated (but realistic) situations.

While some fields have many papers that fall into category (2), including clinical decision making, there are very few in the epidemiologic literature.

As a fundamental challenge for causal inference and a potential source of error in all observational study results, confounding is, and perhaps will always be, a thorn in the side of observational epidemiology [12]. Papers that fall into category (3) continue to be published in the epidemiologic and statistical literature [13,14], but no completely satisfying method has emerged.

Questions of how to think about, and quantify the effect of, random error in nonrandomized studies remain unresolved. A standard analysis pretends that researchers or nature randomized the study exposure to study subjects [15,10]. Is this the best we can do?

Study results are a function of both the data being analyzed and assumptions made in the process of the analysis [16-18]. But surprisingly little attention has been paid in the epidemiologic literature to category (5), the impact of incorrect statistical assumptions on study results. Vandenbroucke's [19] question, "Should we abandon statistical modeling altogether?" and Greenland's [10] question, "Are conventional statistics anything other than misleading?" deserve serious attention.

## Philosophies of the science

There is a substantial amount of epistemologic discussion in epidemiology, mostly relating to how we know if we are seeing a causal relationship and, to a lesser extent, what causation means. We hope that scientists in the field will become more actively engaged in practical epistemology (and learn to recognize such inquiry as valid scholarly analysis, rather than commentary), and we invite them to submit their work to *EP&I*. Epistemology in health science is often treated as a matter of statistical rules of thumb, with litigation serving as the ultimate arbiter. Neither statistical rules nor the results of lawsuits that go to trial are a particularly good source of knowledge.

Also falling under the broad category of philosophy is analysis of ethics. In the health sciences, such discussion is dominated by the issue of protecting human research subjects and patients. What is largely missing, and what we would like to invite submissions about, is the ethics of the core of our science: study design, data analysis, and result reporting. Since it is impossible to report every result that might be gleaned from a dataset, what constitutes an ethical choice of what to present? This question is central to most everything that most epidemiologists do, and yet there is remarkably little discussion about it [20]. How many results need to be presented to justify the social expense of doing the research? Should we consider unethical the reporting of results in ways that make them seem larger or more important?

Many epidemiologists consider the field *per se *to include policy advocacy based on health science findings. Whether or not one takes that position, discussions of and about epidemiology should address questions of what society ought to do with our findings. (If we do not bring careful analysis to such questions, who will?) One interesting and timely set of questions involves the relative merits of decision-analysis or risk-analysis based decisions, "precautionary-principle" based decisions, and actual current practices.

## Exemplars, reconsiderations, and debates

We seek to acknowledge the best epidemiologic studies and papers with articles that discuss what sets these studies apart from the rest: a "Best of Epidemiology" series. Much of methods training involves finding the worst in a study. We believe it would be useful to give students and other epidemiologists examples of how to do epidemiology well. What studies should we most want to emulate, and why?

In a similar vein, we hope to receive submissions that remind us about the historical foundations of epidemiology: a "History of Epidemiology" series. What historical studies, insights, or methodology are important for understanding the field, and why, and which are simply interesting enough to be worth highlighting? History-of-science type analysis of our field, almost completely absent from the history-and-philosophy-of-science literature, would be particularly welcome.

A complement to the "best of" and recounting of historical high-points is reanalysis of particular study results or aspects of the field's literature more generally. Many substantive letters to the editor about published papers reflect a full article's worth of analysis by the letter authors. But those authors have little opportunity to publish that analysis, even when it has substantial practical or methodological importance; we encourage submission to *EP&I *of such papers, whether or not a letter to the editor has been published. We encourage authors to take advantage of the speed of online publishing to submit re-analyses of important findings for publication while the implications of the original study are still being debated. (Important re-analyses, based on either the original data or sometimes merely what was published, need not be immediate, however, and can still be interesting years later.) Readers of the health science literature may often find themselves asking a question about a study's results beyond the few specifically addressed in the published reports. A new analysis might be more relevant to a particular decision, or even out-and-out more informative than the original publication.

Finally, we hope to publish "Point-Counterpoint" collections on single topics. Authors considering writing a counterpoint (or simply a related point) to an *EP&I *article are encouraged to contact us in advance to see if we might solicit other related submissions to create a larger collection. Topics of particular interest to us, where we would like to encourage the starting of point-counterpoint series, include the usefulness of causal inference in epidemiology and the scientific basis for a particular regulatory decision (e.g., the U.S. Food and Drug Administration decisions to ban phenylpropanolamine and ephedra). We especially encourage students to suggest potential topics.

## Wishful wish list?

We will grant that this is an ambitious research agenda. Perhaps it will be years before journals (this one or others) see articles on all these topics. But it is also possible that the next 100 dissertations in the field will include chapters that cover most of this ground. And perhaps those of us well past our dissertations can contribute a few also. We hope that asking (and providing a venue for publication) will make this much more likely.
